# Editorial: Cancer and Bone Metastasis

**DOI:** 10.3389/fendo.2019.00852

**Published:** 2019-12-04

**Authors:** Chandi C. Mandal

**Affiliations:** Department of Biochemistry, Central University of Rajasthan, Ajmer, India

**Keywords:** cancer, microenvironment, bone metastases, osteolysis, stromal

## Introduction

In spite of great efforts, cancer continues to be a leading cause of mortality and morbidity nationwide. Recent advances through innovation and discoveries in cancer biology have largely improved overall survival as well as quality of life in cancer patients. This is through the treatment of primary tumors, when the tumors are diagnosed at an early stage. The development of cancer metastases is still a deadly threat to cancer patients. Metastases frequently occur in bone tissue since it provides fertile nutrients to those disseminated tumor cells which had already traveled to bone environment. Bone is an active and dynamic tissue which is continuously remodeled by balanced and coordinated action of bone forming osteoblast and bone resorbing osteoclast cells. Dispersed tumor cells growing in the bone microenvironment often supply various cytokines/chemokines to bone resident cells. This eventually disrupts the balanced action between these two types of bone resident cells. Here, excess osteoclast activity leads to develop osteolytic lesions, whereas abnormal osteoblast activity drives to develop osteoblastic metastases. Indeed, cancer cells derive various cytokines (e.g., CSF-1, RANKL, DKK-1, JAGGED 1, etc.) either directly or indirectly which promote bone metastasis. Thus, the detailed mechanism for understanding the influence of bone microenvironment and adjacent stromal cells in the development of metastases is of urgent need. Understanding this process might identify targets in which one could design therapies for metastatic bone disease. Articles published on the topic “cancer and bone metastasis” have described important mechanisms and cellular interaction involved in bone metastasis.

## Interaction Between Cells at Metastatic Niche

Osteolytic metastasis increases fracture risk and leads to develop cachexia. Guise TA research group described herein that this osteolytic metastasis also causes skeletal muscle weakness (Regan et al.). Increased oxidative stress caused by disseminated cancer cells might accelerate the pathological process of the sarcoplasmic Ca^++^ release from muscle cells to make the muscle weak. This would further potentiate fracture risk. In depth studies have found an involvement of TGF-β/NOX4/RyR1 signaling in breast cancer osteolyitc induced muscle weakness. In fact, disseminated cancer cells present in bone environment disrupts bone remodeling by altering the activity of osteoblast and osteoclast cells. Mechanical loading prevents bone metastasis. Lynch ME research work suggested that in bone metastasis, mechanical loading increases osteocyte dendrite formation and downstream resorption (Wang et al.). This study further suggested that loading condition might increase and/or alter soluble factors (which are yet to be identified) to enhance osteocyte E11 expression and remodeling RANKL/OPG ratio along with decreasing osteocyte cells. Beside bone cells, various stromal cells including immune, endothelial, fibroblast, and adipocytes (directly or indirectly) modulate survival, dormancy, growth of disseminated cancer cells, and metastatic activity by supplying various factors and modulating intracellular signals, in addition to cell-cell interaction. Lynch research team highlighted the impact of bone resident macrophages on bone metastasis and cancer cell progression at metastatic sties. The factors CSF-1 and CCL2 released by disseminated cancer cells, recruit macrophages to the metastatic environment (Lo and Lynch). However, these recruited macrophages may polarize into pro-inflammatory and/or anti-inflammatory depending on the molecular and cellular components present at the metastatic niche. These polarized macrophages seem to have various roles in cancer progression and osteoblast/osteoclast activity. Uma Sankar research group also emphasized the recruitment of macrophages to the site of bone metastases by prostate cancer cells (Dadwal et al.).

## Cellular Signaling for Target Identification and Therapy

Dadwal et al. outlined how androgen-deprivation therapy (ADT) not only affects bone health, but promote cancer resistance probably because of mutations in the androgen receptor (AR). Such mutations activate downstream CaMKK2 signaling even in the presence of very low androgen levels. Thus, targeting AR-CaMKK2 seems to be a therapeutic strategy for metastatic bone disease. Similarly, Suvannasankha group pointed out that blocking semaphoring 4D (Sema4D) may prevent osteolytic deposits along with inhibition of cancer progression both at the primary and metastatic sites (Lontos et al.). Both Sema4D and its receptor, Plexin B1 are often deregulated in various cancers. In addition, Sema4D expressed in mature osteoclast binds to Plexin B1 present on the osteoblast cell surface. This receptor ligand interaction not only inhibits osteoblast differentiation, but it also promotes angiogenesis. Galson DL research team reported that small molecule inhibitor, XRK3F2, reduced osteoclast activity along with the suppression of multiple myeloma (MM) growth (Adamik et al.). Moreover, this inhibitor blocks P62-ZZ domain signaling to rescue MM-suppressed osteoblast differentiation by reducing the transcriptional epigenetic repressor of RunX2, a key osteoblast differentiation factor. Martin TJ research study suggests that PTHrP might work differentially in disseminated cancer cells, and in bone osteoblast/osteocyte cells to promote metastases (Johnson et al.). They suggest that PTHrP potentiates the growth of cancer cells at the site of osteolytic deposits by reducing expression of tumor dormancy genes presumably via PTHR1/cyclic AMP-independent manner; whereas it can alter bone homeostasis by acting with osteoblast/osteocyte cells following canonical PTHR1/cyclic AMP signaling pathway.

The bone microenvironment also modulates the level of various microRNAs and could be targeted by specific metastatic therapy. In this context, Haider MT research group emphasized the role of various microRNAs in regulation of metastatic growth (Haider and Taipaleenmaki). For example; miR-34a, miR-133a, miR-141, and miR-219 inhibit osteoclast activity, whereas miR-135 and miR-203 prevent macrometastases. Similarly, miR-218 and miR-296 increase osteoblast and osteoclast activity, respectively. Thus, delivery of these microRNAs or antagomiRs can be used to limit disease progression in metastatic bone disease. Similarly, Reagah MR research team suggested that the use of proteasome inhibitors (PI) in controlling multiple myeloma induced bone disease will be more effective if PI resistance is overcome (Farrell and Reagan).

## Advanced Imaging and Surgical Perspective for Metastatic Bone Disease

However, it is quite difficult to diagnose metastatic bone disease at an early stage. Silbermann R group suggested that various advanced imaging techniques including (i) skeletal survey (SS), (ii) whole body computed tomography (WBCT), and (iii) positron emission tomography-CT (PET-CT) could be used for the detection of very small sized osteolytic lesions. They also emphasized the improved combination of WBCT and 18F fluoro-deoxyglucose (FDG) PET-CT for visualizing bone marrow infiltration along with whole body tumor burden (Hansford and Silbermann). Similarly, both surgical and non-surgical multimodal treatment strategies are required to deal with metastatic bone disease. Choong PF research group emphasized that orthopedic surgeons have a critical role in the decision making to choose the most effective treatment strategy. This may depend on the site (e.g., femur, humerus, etc.) and bone metastasis type (e.g., breast, lung, etc.), since type of implants and surgical options available will influence the outcome (Soeharno et al.).

## Conclusion

In brief, the articles published on the Research Topic “cancer and bone metastasis” emphasized the importance of various molecules, different cell types, and crucial signaling pathways working in various bone metastatic niches, and how they influence our understanding of metastatic bone disease. This is in addition to advancing imaging diagnostic techniques and various surgical options in dealing with such devastating disease. The Research Topic also suggested various non-surgical treatment options including proteasome inhibitors, small molecule inhibitors and microRNAs. Being an editor, I would like to express my gratitude to all the contributing authors for providing valuable research manuscripts to this Research Topic.

## Author Contributions

CM has formulated and written the editorial.

### Conflict of Interest

The author declares that the research was conducted in the absence of any commercial or financial relationships that could be construed as a potential conflict of interest.

